# Supplementary material for: The Periaqueductal Gray Selectively Supports Reversal Learning During a Flexible Discrimination Task in Mice
Source: bioRxiv. 2026 Apr 1:2026.01.19.700312. Originally published 2026 Jan 19. Preprint. [Version 2] doi: 10.64898/2026.01.19.700312 (PMC12871592; doi:10.64898/2026.01.19.700312)
Supplement: 1 [file NIHPP2026.01.19.700312v2-supplement-1.pdf]

bioRxiv preprint doi: https://doi.org/10.64898/2026.01.19.700312; this version posted April 1, 2026. The copyright holder for this preprint (which was not certified by peer review) is the author/funder, who has granted bioRxiv a license to display the preprint in perpetuity. It is made available under aCC-BY 4.0 International license.

Supplementary Materials

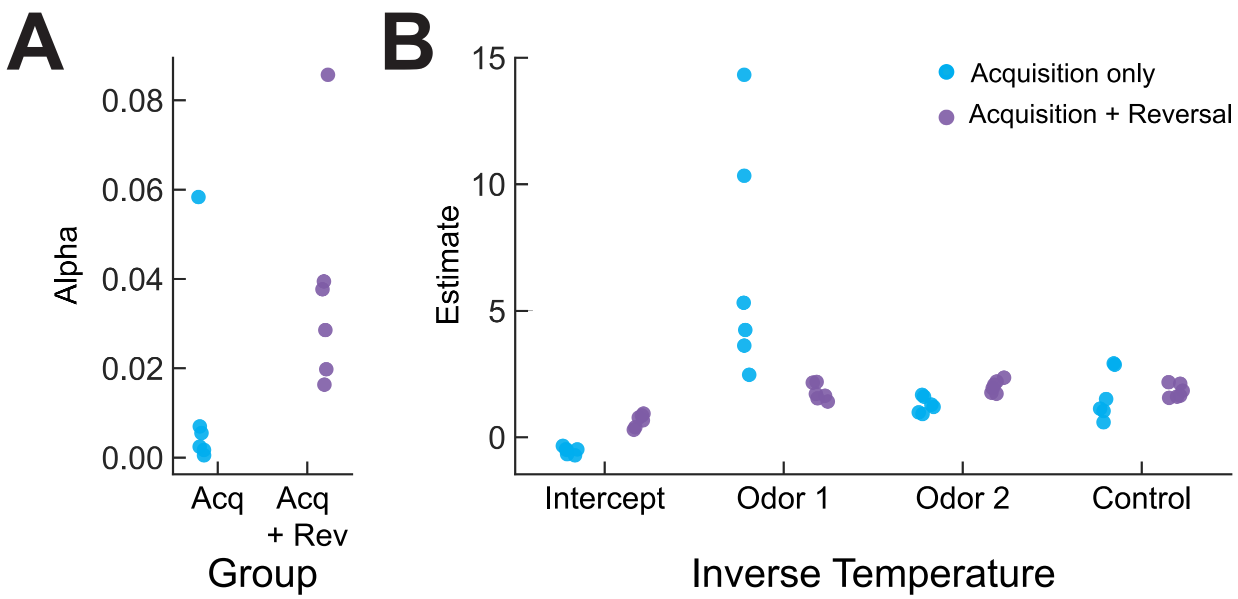

Supplementary Figure 1. Group parameters computed by the Q-learning algorithm. Fitting parameters are shown for the two modeling groups: acquisition only (*blue*) and acquisition following reversal (*purple*; $n = 6$ per group). **(A)** Learning rate parameter alpha. **(B)** Beta estimates for intercept (bias to lick), odor 1 (go stimulus), odor 2 (no-go stimulus) and control for changes in airflow during final valve opening (nitrogen).

bioRxiv preprint doi: https://doi.org/10.64898/2026.01.19.700312; this version posted April 1, 2026. The copyright holder for this preprint (which was not certified by peer review) is the author/funder, who has granted bioRxiv a license to display the preprint in perpetuity. It is made available under aCC-BY 4.0 International license.

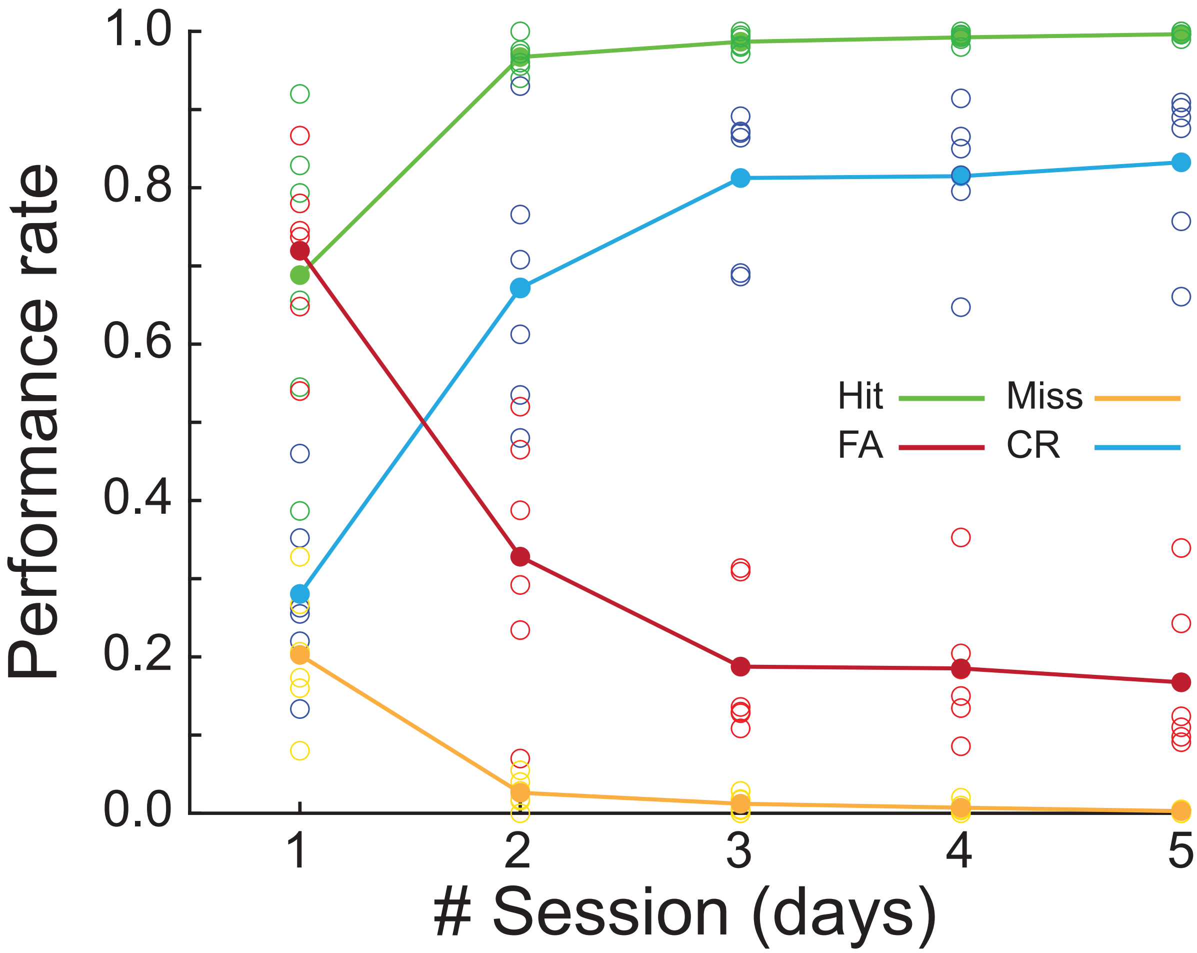

Supplementary Figure 2. Group performance for the different discrimination conditions during Reversal. Behavioral performance rates across five consecutive sessions during the Reversal phase divided by the lick response of the animal to each cue type: Hit (lick to go odor; *green*), Miss (no lick to go odor; *yellow*), False Alarm (FA, lick to no-go odor; *red*) and Correct Rejection (CR, no lick to no-go odor; *light blue*). Filled circles indicate group mean and open circles indicate individual subjects (n = 6).

bioRxiv preprint doi: https://doi.org/10.64898/2026.01.19.700312; this version posted April 1, 2026. The copyright holder for this preprint (which was not certified by peer review) is the author/funder, who has granted bioRxiv a license to display the preprint in perpetuity. It is made available under aCC-BY 4.0 International license.

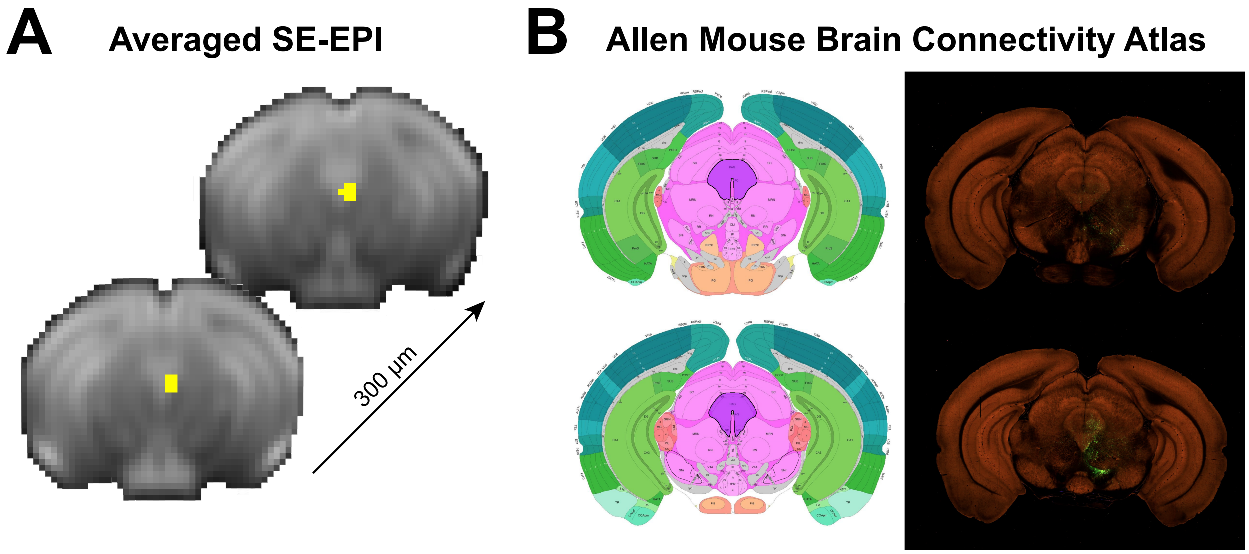

Supplementary Figure 3. Spatial localization of the periaqueductal gray across fMRI and atlas space. (A) Periaqueductal gray mask (yellow) defined based on regions showing a significant BOLD response in the whole-brain analysis and further used in the ROI analysis. The ROI mask is presented on an average raw fMRI data (spin-echo echo planar imaging), shown as sequential coronal slices with a slice thickness of 300 µm. **(B)** Coronal images (*left*, atlas; *right*, two-photon tomography) taken from the Allen mouse brain connectivity atlas that correspond to the spatial location of the fMRI data. The region highlighted in purple denotes the Periaqueductal gray as defined by the Allen Institute. Image identification numbers are 87 (bottom) and 90 (top) in the reference atlas.

Supplementary Table 1. Summary of number of sessions completed by each subject.

bioRxiv preprint doi: https://doi.org/10.64898/2026.01.19.700312; this version posted April 1, 2026. The copyright holder for this preprint (which was not certified by peer review) is the author/funder, who has granted bioRxiv a license to display the preprint in perpetuity. It is made available under aCC-BY 4.0 International license.

**Subject**	**# Sessions**			
	**Behavior**		**fMRI**	
	Acquisition	*Reversal*	*Acquisition*	*Reversal*
Subject 01	10	—	9	—
Subject 02	10	—	9	—
Subject 03	6	—	6	—
Subject 04	9	5	8	5
Subject 05	15	—	15	—
Subject 06	16	6	14	6
Subject 07	15	6	13	6
Subject 08	14	6	12	6
Subject 09	9	—	9	—
Subject 10	9	—	9	—
Subject 11	8	5	8	5
Subject 12	8	5	8	5

Summary of maximal number of sessions (one session per day) completed by each subject during the *Acquisition* and *Reversal* experimental phases (Behavior). All subjects reached learning criterion in the *Acquisition* phase by the fourth session (3.583 ± 1.621, mean ± SD). Of the 6 subjects participating in the *Reversal* phase, 3 subjects completed 8–9 sessions, and 3 completed 14–16 sessions. This manipulation was used to rule out that an extended number of sessions during *Acquisition* affects the results observed during *Reversal*. The table also shows the number of usable fMRI data (fMRI) session, as some were excluded due to software issues or poor performance inside the scanner.
